# Monitoring the effect of perioperative nutritional care on body composition and functional status in patients with carcinoma of gastrointestinal and hepatobiliary system and pancreas

**DOI:** 10.2478/raon-2023-0028

**Published:** 2023-07-13

**Authors:** Andrej Gyergyek, Nada Rotovnik Kozjek, Jasna Klen

**Affiliations:** Faculty of Medicine, University of Ljubljana, Ljubljana, Slovenia; Department for Clinical Nutrition, Institute of Oncology Ljubljana, Ljubljana, Slovenia; Department of Abdominal Surgery, University Medical Centre Ljubljana, Ljubljana, Slovenia

**Keywords:** abdominal cancer, nutritional status, body composition, oral nutritional supplements, nutritional care

## Abstract

**Background:**

The significance of nutritional care in the management of cancer, particularly in the surgical treatment of abdominal cancer, is increasingly acknowledged. Body composition analysis, such as the Bioelectric impedance assay (BIA), and functional tests, *e.g.,* handgrip strength, are used when assessing nutritional status alongside general and nutritional history, clinical examination, and laboratory tests. The primary approach in nutritional care is individually adjusted nutritional counselling and the use of medical nutrition, especially oral nutritional supplements. The aim of the study was to investigate the effects of perioperative nutritional care on body composition and functional status in patients with carcinoma of the gastrointestinal tract, hepatobiliary system, and pancreas.

**Patients and methods:**

47 patients were included, 27 received preoperative and postoperative nutritional counselling and oral nutritional supplements (Group 1), while 20, due to surgical or organisational reasons, received nutritional care only postoperatively (Group 2). The effect of nutritional therapy was measured with bioimpedance body composition and handgrip measurements.

**Results:**

Group 2 had a higher average Nutritional Risk Screening (NRS) 2002 score upon enrolment (3 vs. 2 points); however, there was no difference when malnutrition was assessed using Global Leadership in Malnutrition (GLIM) criteria. There was a relative increase in lean body mass and fat-free mass index (FFMI) 7 days after surgery in group 1 (+4,2% vs. −2,1% in group 2). There was no difference in handgrip strength.

**Conclusions:**

Our results indicate that combined preoperative and postoperative nutritional care is superior to only postoperative nutritional care. It seems to prevent statistically significant lean mass loss 7 days after surgery but not after 14 days or 4 weeks.

## Introduction

Cancers of the gastrointestinal tract (colon, stomach, liver, rectum, or oesophagus) (GIT) represent about 23.2% of new cancer cases. Meanwhile, pancreatic and biliary carcinoma are less common and only account for 3.1% percent of cases but are far more lethal and together represent 5.6% of cancer deaths.^1^ With the prevalence of colorectal cancer rapidly increasing around the world, the number of patients who are undergoing surgical procedures as primary treatments is increasing proportionately.^2,3^ Surgery is the mainstay treatment for cancer^4^ and is complemented by chemoand radiotherapy, both pre- and postoperatively.^5^ Malnutrition is often present before the start of the cancer treatment, and its prevalence only increases after the treatment's completion.^6,7^ The correlation between malnutrition, and postoperative complications and mortality has been well documented in prospective and retrospective studies.^8,9,10^ Low muscle mass represents one of the diagnostic criteria for malnutrition. In cancer patients, its loss throughout the course of the disease is not consistent, as catabolic stress, such as surgery, accelerates proteolysis. After the disease stressor is removed, proteolysis subsides to levels before the onset of disease.^11^

The nutritional care process is the basis for the recognition of malnutrition and for the initiation of nutritional support. It starts with nutritional risk screening^12^, where the recommended screening method in hospitals is the Nutritional Risk Screening (NRS) 2002 survey. Patients who are found to be at nutritional risk require a complete assessment of nutritional status in which a combination of objective and subjective parameters should be utilised.^13^ For a definitive diagnosis of malnutrition, the Global Leadership in Malnutrition (GLIM) criteria are used.^14^ Low muscle mass, in combination with function decline, leads to sarcopenia^15^, which is linked to detrimental outcomes after surgical treatment of abdominal cancer, indicated by increased readmission rates and worse chemotherapy tolerance.^16^ In clinical practice, the bedside body composition measurement with bioimpedance and the measurement of function with handgrip are frequently used for assessing patients’ nutritional status. These measurements provide valuable information that contributes to the identification, diagnosis, and management of several medical conditions for which nutrition therapy is indicated.^17^ The correlation of body composition with health and functional status is well established.^18,19^ The hand grip strength test is among the most widely used measures of physical ability; lower hand grip strength has been proven to be a good indicator of postoperative complications, longer hospitalisations, and worse physical status. It is also an excellent prognostic factor of both short- and long-term mortality.^20^

Once a patient is found to be at risk for nutritional deficiency, treatment should be initiated. Oral nutritional supplements (ONS) are considered the first choice for nutritional treatment, along with enteral nutrition. Additionally, patients should be counselled about eating their usual diet until the night before the surgery and the correct use of ONS.^8^ If an elective surgical patient is malnourished, the appropriate nutritional therapy should be implemented, and non-emergency surgeries postponed. ONS are recommended for use in all malnourished cancer patients and all high-risk patients for abdominal surgery.^8^ In a recent meta-analysis, perioperative nutritional supplementation has been shown to decrease postoperative infectious and non-infectious complications and length of stay in patients undergoing gastrointestinal cancer surgery.^21^

In our pilot study, we analysed two different nutritional care approaches in our clinical practice to determine if patients receiving both preoperative and postoperative nutritional care have better body composition and functional status after surgery compared to the group that only received postoperative nutritional care. For body composition, we focused on the assessment of lean mass with fat-free mass index (FFMI), 3^rd^ space water, and phase angle. We expected the changes in body composition to be reflected in functional status and clinical course of the treatment.

## Patients and methods

### Study design and population

This prospective observational study was conducted between October 2021 and May 2022 at the Department of Abdominal Surgery of University Medical Centre (UMC) Ljubljana and at the clinical nutrition unit at the outpatient clinic of UMC Ljubljana. The committee for medical ethics of the Republic of Slovenia approved the study (permit number 020-427/2021/6). The Declaration of Helsinki, The Council of Europe Oviedo Convention and its protocols were followed, and all patients signed informed consent forms. They were all treated according to the established clinical guidelines and principles of good clinical practice.

Patients with carcinoma of GIT and hepatobiliary tract and pancreas were randomly enrolled in the study during their preoperative appointment if they were above 18 years of age and were to undergo surgical treatment of carcinoma of either GIT, hepatobiliary system, or pancreas. Group 1 (G1) patients were included into the study by being invited into the clinical nutrition outpatient clinic after their preoperative appointment with the anaesthesiologist. It was at this point that they started taking ONS preoperatively. ONS, which are immune-modulating formulae consists of the following important components: fish oil (eicosapentaenoic acid [EPA, 0.4g] + docosahexaenoic acid [DHA]), medium chain triglycerides (MCT), vitamins D3, C, A, K, B2, 6, 12, essential elements, inulin, maltodextrin and sucrose. The patients took ONS for 7 days, after which they had their surgery. Group 2 (G2) patients were included upon admittance to the surgical ward the day before surgery, where their physical status and treatment plan allowed for immediate surgery. Therefore, the patients in G2 were called in for surgery just days after their preoperative appointment, and there was no time for preoperative nutritional preparation.

Patients were considered ineligible to participate if: they had taken ONS before being included in the study or were already being followed in another clinical nutrition unit; the carcinoma was not histologically confirmed; they withdrew their consent at any point during the study; they were taking or had previously taken illicit drugs; they had a mental health disorder that prevented them from understanding and following nutritional treatment; their participation in the study would cause them far greater harm and risk than the potential benefits (*i.e.* due to old age or numerous associated diseases).

### Data collection

Data for G1 were collected at their appointment in the outpatient clinic, where general and nutritional history were assessed, body composition was measured, and handgrip strength was measured using Jamar handheld digital dynamometer (Jamar Plus Digital, Performance Health, IL, USA). In G1 no measurements were made the day before or the morning of operative procedure. The data collection for G2 started at admission to the surgical ward or the morning before the surgical procedure. Handgrip strength (kg) was not measured in the G2 at the enrolment into the study. In both groups, anthropometric data were measured, and body composition was analysed using the bioelectric impedance assay (BIA) method with BodyStat Quadscan 4000 Touch device (Bodystat, Isle of Man, UK), FFMI was calculated by BIA device using its own algorithms and nutritional risk screening was performed using the NRS 2002. We compared body mass (kg), body mass index (BMI, kg/m^2^), lean mass (kg), FFMI (kg/m^2^), phase angle (⁰), 3^rd^ space water (litres), NRS 2002 score (points), and the percentage (%) of malnourished patients according to GLIM criteria. BIA is a non-invasive and simple method for measuring body composition based on calculations from measuring the electrical conductivity of the body for one or more electric currents.^22^ The body composition measurement 7 days post-surgery, while patients were still staying in the hospital, was used in the study (second measurement).

All patients were invited to two follow-up checks at the outpatient clinic. The third measurement was on the 14^th^ day post-surgery, at which time all patients were already back home and could tolerate oral intake, including ONS. The final check-up was at 4 weeks after surgery (fourth measurement). Nutritional monitoring was performed, ONS compliance was checked and they received nutritional counselling from a clinical dietician. A physician specializing in clinical nutrition supervised nutritional monitoring to plan an individually adjusted treatment. Patients were given verbal and written instructions about nutritional therapy.

### Statistical analysis

In statistical analysis, the variables were first characterised using descriptive statistics, using frequencies for categorical variables, and median with 25%–75% range for continuous variables as not all variables were normally distributed. Data were analysed on an intention-to-treat basis, with all patients remaining in their original allocated group for all analyses. Based on our sample size and the distribution of the variables, we were able to detect a difference between groups of approximately 2.3 for FFMI and 0.74⁰ for phase angle with 80% power. Power analysis was performed using Power and Sample Size Calculation version 3.0.43. For comparison between groups G1 and G2, Fisher's exact test was used for categorical variables and the Mann-Whitney test for continuous variables, including relative change between two time points. For comparison of continuous measurements obtained at different time points, Wilcoxon's test for related samples was used. The cut-off for statistical significance was considered to be p<0.05. All statistical analyses were performed using IBM SPSS Statistics, version 27.0 (IBM Corporation, Armonk, NY, USA).

## Results

### Clinical characteristics of participants at different time points in the study

During the course of our prospective study, data was collected for 47 patients. They were, on average, 72 years old and predominantly male. The distribution of cancer diagnoses among the patients shows that GIT tumours were slightly more prevalent, while the remainder of the cases consisted of various types of liver, gallbladder, biliary system, or pancreatic cancer. Table 1 describes the baseline demographic and clinical characteristics of participants.

**TABLE 1.** Baseline demographic and clinical characteristics of patients (n = 47)

**Variable**		**All participants** **N = 47**	**G1** **N = 27**	**G2** **N = 20**	**P**
Sex	Male, N (%)	33 (70.2)	21 (77.8)	12 (60.0)	0.214
	Female, N (%)	14 (29.8)	6 (22.2)	8 (40.0)	
Age	Years, mean ± SD	70.5 ± 11.2	70.3 ± 8.7	70.7 ± 12.9	0.477
Diagnosis	GIT tumours, N (%)	27 (57.4)	15 (55.6)	12 (60.0)	1.000
	Tumours of liver, gallbladder, biliary system and pancreas, N (%)	20 (42.6)	12 (44.4)	8 (40.0)	

GIT = gastrointestinal tract; N 0 number; SD = standard deviation

Upon enrolment, the participants in G1 and G2 had no significant differences in any of the variables measured. Clinical characteristics of all patients are represented in Table 2.

**TABLE 2.** Clinical characteristics of patients upon enrolment into the study

**Variable**		**G1** **N = 27**	**G2** **N = 20**	**P**
Body mass	kg, median (25–75%)	82.0 (70.0–98.0)	78.5 (72.3–88.3)	0.268
BMI	kg/m^2^, median (25–75%)	26.2 (23.4–34.5)	27.5 (24.3–29.6)	0.569
Lean mass	kg, median (25–75%)	53.1 (47.1–64.2)	53.9 (45.0–59.6)	0.505
FFMI	median (25–75%)	17.8 (16.4–20.3)	17.6 (15.9–20.4)	0.561
Phase angle	°, median (25–75%)	4.7 (4.3–5.4)	4.6 (4.0–5.5)	0.846
3^rd^ space water	L, median (25–75%)	−0.1 (−0.8–1.0)	0.4 (−0.4–0.9)	0.425
NRS 2002	Points, median (25–75%)	2 (0–3)	3 (3–3,8)	0.012
Malnutrition according to GLIM	No, N (%)	14 (51.9)	16 (80.0)	0.067
	Yes, N (%)	13 (48.1)	4 (20.0)	

BMI = body mass index; FFMI = fat free mass index; GLIM = Global Leadership in Malnutrition

**FIGURE 1.**
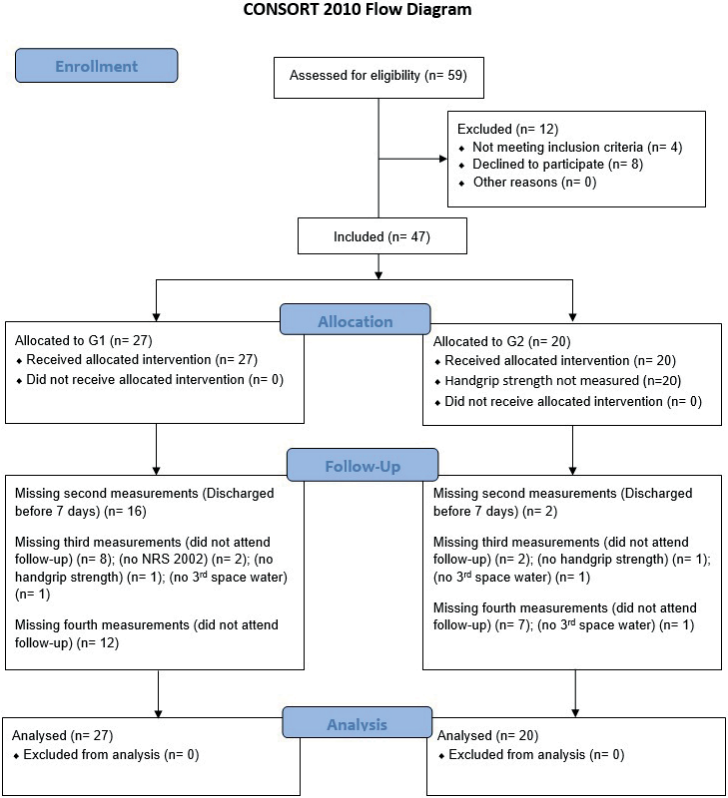
Consolidated Standards of Reporting Trials (CONSORT) flow diagram.

There were no significant differences in absolute values of measured variables between G1 and G2 at any point during the study. The data is summarised in Table 3 for handgrip strength and in Table 4 for anthropometric and body composition analysis as well as NRS 2002 score. There were several missing measurements at different points during the study because the protocol was not followed, as is summarised in Figure 1.

**TABLE 3.** Handgrip strength data

**Variable**		**Upon enrolment**		**After 7 days**	**After 14 days**			**After 4 weeks**		

		**G1** **N = 27**	**G2**		**G1** **N = 19 [8]**	**G2** **N = 18 [2]**	**P**	**G1** **N = 15 [12]**	**G2** **N = 13 [7]**	**P**
Hand grip strength	kg, median (25–75%)	34.1 (28.5–41.6)	/	/	30.2 (25–35.3)	30.3 (25.8–36.8)	0.782	29.7 (23.6–32.7)	32.6 (27.3–35.6)	0.254
Hand grip strength: norm	No, N (%)	19 (70.4)	/		11 (61.1)	9 (52.9)	0.738	8 (53.3)	8 (61.5)	0.718
	Yes, N (%)	8 (29.6)	/		8 (47.1)	7 (38.9)		7 (46.7)	5 (38.5)	
Hand grip strength: deviation	median (25–75%)	0.2 (−0.1–1.2)	/		0 (−0.5–0.5)	0.3 (−0.4–1)	0.369	0.1 (−0.7–0.3)	0.3 (−0.8–.8)	0.683

norm = patients meets the norm for hand grip strength for age and sex; ^**^Hand grip strength: deviation = deviation of hand grip strength from the norm expressed as a multiple of standard deviation

[number of missing participants]

**TABLE 4.** Clinical characteristics of patients at different time points

**Variable**		**After 7 days**			**After 14 days**			**After 4 weeks**		

		**G1** **N = 11 [16]**	**G2** **N = 18 [2]**	**P**	**G1** **N = 19 [8]**	**G2** **N = 18 [2]**	**P**	**G1** **N = 15 [12]**	**G2** **N = 13 [7]**	**P**
Body mass	kg median (25–75%)	72.0 (67.8–93.2)	76.4 (68.5–85.6)	0.912	73.0 (65.0–92.0)	75.5 (70–84.5)	0.869	69.4 (66–76.8)	75 (72.5–86.5)	0.130
BMI	kg/m^2^ median (25–75%)	25.4 (22.7–36.1)	26.8 (24.1–29.0)	0.808	25 (22.5–34.1)	26.4 (22–28.9)	0.620	23.8 (21.7–27.2)	26 (23–29.5)	0.413
Lean mass	Kg median (25–75%)	52.0 (48.2–53.2)	52.0 (41.9–57.1)	0.842	50.1 (46.4–52.1)	53.2 (45–58)	0.707	48.4 (41.7–50.6)	55 (46.5–60.2)	0.065
FFMI	median (25–75%)	17.8 (16.5–20.8)	17.4 (15.8–19.0)	0.340	16.8 (15.8–19.4)	17.4 (15.6–19.6)	1.000	16.5 (15.1–17.5)	18.8 (15.9–19.8)	0.118
Phase angle	median (25–75%)	4.3 (3.5–4.9)	4.4 (3.3–5.1)	0.947	4.4 (4.1–5)	4.2 (3.6–4.7)	0.461	4.8 (3.7–5.3)	4.4 (3.3–4.7)	0.201
3^rd^ space water	L median (25–75%)	0.7 (−0.3–1.6)	0.4(−0.6–1.0)	0.642	0.5 (−0.–1.4)	0.2 (−0.1–1)	0.988	0.4 (−0.2 do 1.1)	0.4 (−0.2–1.3)	0.928
NRS 2002^**^	Points median (25–75%)	/			4 (3–4.3)	4 (3–4.5)	0.961	4 (3–5)	3 (3–4)	0.235

BMI = body mass index; FFMI = fat free mass index; NRS 2002 = score achieved on screening with NRS 2002 tool

[number of missing participants]

### Relative differences between the two groups when compared to the starting values

We found a significant difference in lean mass and FFMI after 7 days, where the G1 lean mass and FFMI increased, and the G2 decreased. The analysis for the third and fourth measurements showed no significant differences between measured parameters (Supplementary Tables 6, 7, and 8).

## Discussion

The results of our pilot study of clinical practice in the Department of Abdominal Surgery of UMC Ljubljana indicate that the combination of pre- and postoperative nutritional care in abdominal cancer patients is superior to only postoperative nutritional care. We found that perioperative nutritional care does seem to prevent statistically significant lean mass loss 7 days after surgery but not after 14 days or 4 weeks. While there has been previous research conducted on the impact of nutritional status in cancer patients and their body composition and functional status^8,9,10,23^, this is the first research in our clinic of patients with carcinoma of GIT, and the first Slovenian study in patients with carcinoma of hepatobiliary system and pancreas.

We checked the nutritional risk score with NRS 2002 and malnutrition according to GLIM criteria. It was crucial to check whether there were significant differences between the two groups at the beginning of the study to see if these differences could have remained present throughout the observation period and affect our findings.

The only significant difference was that patients in group 2 had a 1 point higher average NRS 2002 score, which, although statistically significant, is of questionable clinical importance as it was not reflected in body composition or functional status.

Upon enrolment, all patients were hemodynamically stable and had no clinical signs of malnutrition (*i.e*., oedema, angular stomatitis, improper healing of the wounds, etc.), and reported no history of nutritional disorders. No participants underwent emergency surgery due to ileus or bowel perforation. This is also the case in other studies investigating nutritional care in abdominal cancers, as they are all performed on elective surgical patients.^24,25,26,27^

There were few differences between the observed groups during the course of our study. The main reason could be that the observation time was relatively short, and the number of patients was small. In a recent randomized control trial on patients with colorectal carcinoma, there was an almost 2 points higher skeletal muscle index, lower sarcopenia prevalence, and improved chemotherapy tolerance after 3 months in the intervention group.^27^ Only those with a score of 3 or more points on NRS 2002 upon discharge from the hospital were included. The nutritional risk of patients in their study was estimated to be higher than in ours, which could further explain the lack of significant difference between our G1 and G2 groups, where the minimal NRS 2002 value in their study was 3. Similarly, a study conducted on patients with oesophageal carcinoma found significantly smaller relative muscle mass loss after 3 and 6 months but not after 1 month in patients that received ONS alongside disease state-specific nutrition.^28^ The last check-up in our study was scheduled for 4 weeks after surgery when we expected to detect any change due to the variation in preoperative nutrition. Secondly, this was the time that some of the patients would start postoperative chemo- or radiotherapy, which could have affected the study results.^6,7^ A possible conclusion could be drawn from this that the effects of nutritional care are seen in the longer term, rather than short-term (*i.e.,* 4 weeks post-surgery).

To reduce the variables in our study, all patients took the same type of ONS, EPA containing, which is considered to have an immunomodulatory effect.^29^ A study published in 2019, used the same type of ONS as we did, where patients in the intervention group took ONS for the 7 consecutive days before surgery and for 21 days from when they could again tolerate oral intake.^28^ The average lean mass loss in the control group was 6.74% and 6.89% in the intervention group, and the difference was neither statistically nor clinically significant. The loss was greater than in our study, even though their patients were, on average, 7 years younger. Interestingly, the effectiveness of ONS with EPA in that study was only significant when comparing the data for a subgroup of younger patients. This can be explained by either statistical error due to multiple testing or by weaker anabolic response of skeletal muscle in the elderly, which has been well documented in the medical literature.^29^ Dividing participants into age groups was not reasonable in our study due to the small number of participants.

The importance of measuring body composition and muscle mass can be seen when looking at the second measurement; although there was no noteworthy distinction in body mass or BMI, a marked dissimilarity was observed in lean mass and FFMI. Rinninella *et al*.^30^ found no difference in body mass between intervention and control groups 8 days and 1 month after surgery, on par with our findings based on body mass. Nevertheless, this same study found a significant increase in body mass when using ONS enriched with omega-3 fatty acids. Interestingly, three studies in the aforementioned meta-analysis that looked at muscle mass as opposed to just body mass found no significant difference in neither body mass nor muscle mass.^30^ In addition, the patients received only postoperative ONS, and the control group did not receive ONS at all. Considering this, our results indicate that providing preoperative ONS on top of postoperative ONS might offer additional benefits in the first week after surgery.

When looking at hand grip strength and functional status, there is no difference between the two groups. This can partially be explained by the aforementioned anabolic muscle response as our patients were on average 72 years old. Moreover, there was no controlled exercise regime for patients in our study, although they were encouraged to exercise by the dietitian and clinical nutrition physician. It is well established that better results are achieved when treating malnourished patients and combining ONS with exercise regimens.^31^ Hence, the prehabilitation should be trimodal and include nutrition, physical exercise, and a stress-reducing psychological component.^32^

It is worth highlighting that we did not observe any significant difference between the two groups at the end of the four-week period. Even though the percentage of malnourished patients in the first group was twice that in the second group, it only approached the cut-off for statistical significance. This further supports the previously proposed idea that pre-, in addition to, postoperative ONS might offer further benefits in the first week after surgery, as well as later on during the cancer treatment. This is not a definitive conclusion, but it provides outlines for further research. On top of that, the participants in the second group had a higher average NRS 2002 score but a lower relative share of malnourished patients. This demonstrates that NRS 2002 is a *screening* tool and should not be used for making definitive diagnoses, as it has been previously well established.^8,13^

It is important to address the limitations of our study. Primarily, the number of included patients was relatively low. Since there is no established clinical pathway for nutritional care, the dropout rate was relatively high. In addition to that, the patients that did not attend the follow-ups were predominantly the elderly who lived far away from the clinical nutrition unit. They had a large number of medical appointments postoperatively, and because they perceived nutritional care as less important, they decided not to attend the follow-ups so as to not burden their caretakers (*i.e.,* relatives) with transportation to and from the outpatient clinic. Secondly, the included patients were not truly randomised and this has most likely impacted the results, as the patients who were able to undergo surgery on a short notice were generally relatively fit. The final limitation is that we did not measure any clinical course parameters such as length of stay or quality of life.

In conclusion, there is an indication that combined preoperative and postoperative nutritional care could offer some advantages when compared to only postoperative nutritional care. The findings of this pilot study will be the foundation for establishing a clinical pathway for nutritional care for abdominal cancer patients to positively affect their treatment outcomes at the UMC Ljubljana Department of Abdominal Surgery.

## Supplementary Material

Supplementary Material Details
