# Supplementary material for: Monitoring the effect of perioperative nutritional care on body composition and functional status in patients with carcinoma of gastrointestinal and hepatobiliary system and pancreas
Source: Radiol Oncol. 2023 Jul 13;57(3):371–9. doi: 10.2478/raon-2023-0028 (PMC10561066; doi:10.2478/raon-2023-0028)
Supplement: Supplementary file 1 — Supplementary Material Details [file raon-2023-0028_sm.pdf]

Monitoring the effect of perioperative nutritional care on body composition and functional status in patients with carcinoma of gastrointestinal and hepatobiliary system and pancreas

Andrej Gyergyek, Nada Rotovnik Kozjek, Jasna Klen

Radiol Oncol 2023; 57(3): 371-379.

doi: 10.2478/raon-2023-0028

SUPPLEMENTARY TABLE 1. Baseline demographic and clinical characteristics of participants (n = 47)

Variable	All participants N = 47	First group N = 27	Second group N = 20	P
**Gender**				
Male, N (%)	33 (70.2)	21 (77.8)	12 (60.0)	0.214
Female, N (%)	14 (29.8)	6 (22.2)	8 (40.0)	
**Age**, years, median (25–75%)	72 (64–80)	72 (64–82)	72 (65–77)	0.477
**Diagnosis**				
GIT tumours, N (%)	27 (57.4)	15 (55.6)	12 (60.0)	1.000
Tumours of liver, gallbladder,biliary system and pancreas, N (%)	20 (42.6)	12 (44.4)	8 (40.0)	

GIT = Gastrointestinal tract

SUPPLEMENTARY TABLE 2. Clinical characteristics of participants upon enrolment into the study

Variable	First group N = 27	Second group N = 20	P
**Body mass,** kg, median (25–75%)	82.0 (70.0–98.0)	78.5 (72.3–88.3)	0.268
**BMI,** kg/m^2, median (25–75%)	26.2 (23.4–34.5)	27.5 (24.3–29.6)	0.569
**Lean mass,** kg, median (25–75%)	53.1 (47.1–64.2)	53.9 (45.0–59.6)	0.505
**FFMI,** median (25–75%)	17.8 (16.4–20.3)	17.6 (15.9–20.4)	0.561
**Phase angle,** °, median (25–75%)	4.7 (4.3–5.4)	4.6 (4.0–5.5)	0.846
3rd **space water,** L, median (25–75%)	-0.1 (-0.8–1.0)	0.4 (-0.4–0.9)	0.425
**Hand grip strength,** kg, median (25–75%)	34.1 (28.5–41.6)	/	
**Hand grip strength norm**		/	
No, N (%)	19 (70.4)	/	
Yes, N (%)	8 (29.6)	/	
**Hand grip strength:** deviation, median (25–75%)	0.2 (-0.1–1.2)	/	
**Points,** median (25–75%)	2 (0–3)	3 (3–3.8)	0.012
**Malnutrition according to GLIM**			
No, N (%)	14 (51.9)	16 (80.0)	0.067
Yes, N (%)	13 (48.1)	4 (20.0)	

BMI = body mass index; FFMI = fat free mass index; GLIM = Global Leadership in Malnutrition criteria; Hand grip strength norm = patients meets the norm for hand grip strength for age and sex; Hand grip strength deviation = deviation of hand grip strength from the norm expressed as a multiple of standard deviation; NRS = Nutritional Risk Screening

/ = No data for this group

SUPPLEMENTARY TABLE 3. Clinical characteristics of participants after 7 days

Variable	First group N=11 [16]	Second group N=18 [2]	P
**Body mass,** kg, median (25–75%)	72.0 (67.8–93.2)	76.4 (68.5–85.6)	0.912
**BMI,** kg/m^2, median (25–75%)	25.4 (22.7–36.1)	26.8 (24.1–29.0)	0.808
**Lean mass,** kg, median (25–75%)	52.0 (48.2–53.2)	52.0 (41.9–57.1)	0.842
**FFMI,** median (25–75%)	17.8 (16.5–20.8)	17.4 (15.8–19.0)	0.340
**Phase angle,** °, median (25–75%)	4.3 (3.5–4.9)	4.4 (3.3–5.1)	0.947
3rd **space water,** L, median (25–75%)	0.7 (-0.3–1.6)	0.4 (-0.6–1.0)	0.642

BMI = body mass index; FFMI = fat free mass index;

[number of missing participants in each group]

SUPPLEMENTARY TABLE 4. Clinical characteristics of participants after 14 days

Variable	First group N = 19 [8]	Second group N = 18 [2]	P
**Body mass,** kg, median (25–75%)	73.0 (65.0–92.0)	75.5 (70–84.5)	0.869
**BMI,** kg/m^2, median (25–75%)	25 (22.5–34.1)	26.4 (22–28.9)	0.620
**Lean mass,** kg, median (25–75%)	50.1 (46.4–52.1)	53.2 (45–58)	0.707
**FFMI,** median (25–75%)	16.8 (15.8–19.4)	17.4 (15.6–19.6)	1.000
**Phase angle,** °, median (25–75%)	4.4 (4.1–5)	4.2 (3.6–4.7)	0.461
3rd **space water,** L, median (25–75%)	0.5 (-0.4–1.4)	0.2 (-0.1–1)	0.988
**Hand grip strength*,** kg, median (25–75%)	30.2 (25–35.3)	30.3 (25.8–36.8)	0.782
**Hand grip strength norm***			
No, N (%)	11 (61.1)	9 (52.9)	0.738
Yes, N (%)	8 (47.1)	7 (38.9)	
**Hand grip strength deviation*,** median (25–75%)	0.1 (-0.5–0.5)	0.3 (-0.4–1)	0.369
**NRS 2002**,** points, median (25–75%)	4 (3–4.3)	4 (3–4.5)	0.961

BMI = body mass index; FFMI = fat free mass index; Hand grip strength: norm = patients meets the norm for hand grip strength for age and sex; Hand grip strength deviation = deviation of hand grip strength from the norm expressed as a multiple of standard deviation; N 0 number; NRS 2002 = score achieved on screening with Nutritional Risk Screening 2002 tool;

[number of missing participants];

*additional missing data for 1 participant in first and 1 in second group; **additional missing data for 2 participants in first group

SUPPLEMENTARY TABLE 5. Clinical characteristics of participants after 4 weeks

Variable	First group N = 15 [12]	Second group N = 13 [7]	P
**Body mass,** kg, median (25–75%)	69.4 (66–76.8)	75 (72.5–86.5)	0.130
**BMI,** kg/m^2, median (25–75%)	23.8 (21.7–27.2)	26 (23–29.5)	0.413
**Lean mass,** kg, median (25–75%)	48.4 (41.7–50.6)	55 (46.5–60.2)	0.065
**FFMI,** median (25–75%)	16.5 (15.1–17.5)	18.8 (15.9–19.8)	0.118
**Phase angle,** °, median (25–75%)	4.8 (3.7–5.3)	4.4 (3.3–4.7)	0.201
**3rd space water,** L, median (25–75%)	0.4 (-0.2–1.1)	0.4 (-0.2–1.3)	0.928
**Hand grip strength*,** kg, median (25–75%)	29.7 (23.6–32.7)	32.6 (27.3–35.6)	0.254
**Hand grip strength norm***			
No, N (%)	8 (53.3)	8 (61.5)	0.718
Yes, N (%)	7 (46.7)	5 (38.5)	
**Hand grip strength: deviation*,** median (25–75%)	0.1 (-0.7–0.3)	0.3 (-0.8–0.8)	0.683
**NRS 2002**,** points, median (25–75%)	4 (3–5)	3 (3–4)	0.235

BMI = body mass index; FFMI = fat free mass index; Hand grip strength norm = patients meets the norm for hand grip strength for age and sex; Hand grip strength: deviation = deviation of hand grip strength from the norm expressed as a multiple of standard deviation; N = number; NRS 2002 = score achieved on screening with Nutritional Risk Screening 2002 tool;

[number of missing participants].

Relative differences between the two groups when compared to the starting values

SUPPLEMENTARY TABLE 6. Relative changes in clinical characteristics of participants after 7 days

Variable		First group N = 11	Secon group N = 18	P
**Body mass**	%, median (25–75%)	0.0 (-1.9–2.0)	-2.7 (-5.0–0.9)	0.220
**BMI**	%, median (25–75%)	0.0 (-1.9–2.0)	-2.7 (-5.0–0.9)	0.220
**Lean mass**	%, median (25–75%)	4.2 (-1.1–17.3)	-2.1 (-5.5–1.7)	0.039
**FFMI**	%, median (25–75%)	4.3 (-1.3–12.7)	-2.3 (-6.9–1.6)	0.035
**Phase angle**	%, median (25–75%)	0.0 (-23.4–5.4)	-4.4 (-16.9–0.0)	0.774
**3rd space water**	%, median (25–75%)	-22.2 (-100.0–28.6)	11.1 (-48.4–45.8)*	0.342

BMI = body mass index; FFMI = fat free mass index; *data missing for 2 participants

SUPPLEMENTARY TABLE 7. Relative changes in clinical characteristics of participants after 14 days

Variable		First group N = 19	Second group N = 18	P
**Body mass**	%, median (25–75%)	-3.2 (-6.1–0)	-5.6 (-7.9 to -2.5)	0.313
**BMI**	%, median (25–75%)	-3.2 (-6.1–0)	-5.6 (-7.9 to -2.5)	0.313
**Lean mass**	%, median (25–75%)	-2.1 (-7.4–1.2)	-5.5 (-7.5 to -0.6)	0.245
**FFMI**	%, median (25–75%)	-2.5 (-7.4–1.2)	-5.4 (-7.9 to -0.9)	0.245
**Phase angle**	%, median (25–75%)	-6.7 (-7.7–2.1)	-12.3 (-15.6 to -5.1)	0.075
**3rd space water**	%, median (25–75%)	-5.6 (-73.7–103.6)*	-20 (-83.7 to 18.9)*	0.546

BMI = body mass index; FFMI = Fat free mass index; *data missing for 1 participant

SUPPLEMENTARY TABLE 8. Relative changes in clinical characteristics of participants after 4 weeks

Variable		First group N=15	Second group N=13	P
**Body mass**	%, median (25–75%)	-2.8 (-6.9–0.9)	-3.9 (-6.5 to -0.2)	0.821
**BMI**	%, median (25–75%)	-2.8 (-6.9–0.9)	-3.9 (-6.5 to -0.2)	0.821
**Lean mass**	%, median (25–75%)	-2.7 (-8–1.2)	-2.4 (-5.7 to 0.5)	0.856
**FFMI**	%, median (25–75%)	-2.9 (-7.7–0)	-2.3 (-5.5 to 0.4)	0.717
**Phase angle**	%, median (25–75%)	-1.8 (-12.8–9.1)	-9.8 (-18.3 to -5.9)	0.088
**3rd space water**	%, median (25–75%)	-25 (-60–10)	-43.8 (-150.8 to -8.1)*	0.256

BMI = body mass index; FFMI = fat free mass index; *data missing for 1 participant
